# The Svedberg Lecture 2017. From nano to micro: the huge dynamic range of the analytical ultracentrifuge for characterising the sizes, shapes and interactions of molecules and assemblies in Biochemistry and Polymer Science

**DOI:** 10.1007/s00249-018-1321-3

**Published:** 2018-07-28

**Authors:** Stephen E. Harding

**Affiliations:** 1 National Centre for Macromolecular Hydrodynamics (NCMH), School of Biosciences, University of Nottingham, Sutton Bonington, LE12 5RD UK; 2 Kulturhistorisk Museum, Universitetet i Oslo, Postboks 6762, St. Olavs plass, 0130 Oslo, Norway

**Keywords:** Lignin, Amino-cellulose, Vancomycin, Tetanus toxoid, Glycovaccines

## Abstract

The analytical ultracentrifuge (AUC) invented by T. Svedberg has now become an extremely versatile and diverse tool in Biochemistry and Polymer Science for the characterisation of the sizes, shapes and interactions of particles ranging in size from a few nanometres to tens of microns, or in molecular weight, *M* (molar mass) terms from a few hundred daltons to hundreds of megadaltons. We illustrate this diversity by reviewing recent work on (1) small lignin-like isoeugenols of *M* ~ 0.4–0.9 kDa for archaeological wood conservation, (2) protein-like association of a functional amino-cellulose *M *= 3.25 kDa, (3) a small glycopeptide antibiotic (*M *~ 1.5 kDa) and its association with a protein involved in antibiotic resistance (*M *~ 47 kDa), (4) tetanus toxoid protein TTP (*M *~ 150 kDa) and (5) the incorporation of TTP into two huge glycoconjugates considered in glycovaccine development with molecular weight species in a broad distribution appearing to reach 100 MDa. In illustrating the diversity, we will highlight developments in hydrodynamic analysis which have made the AUC such an exciting and important instrument, and point to a potential future development for extending its capability to highly concentrated systems.

## Introduction

It has been an honour for me to have been invited by the Organising Committee to give the 2017 Svedberg Lecture. The analytical ultracentrifuge (AUC) invented by Thé (Théodore) Svedberg has now become an extremely versatile and diverse tool in Biochemistry and Polymer Science for the characterisation of the sizes, shapes and interactions of particles ranging in size from a few nanometres to tens of microns, or in molecular weight (molar mass) terms from a few hundred daltons (g/mol) to hundreds of megadaltons (Scott et al. 2005).

**Fig. 1:**
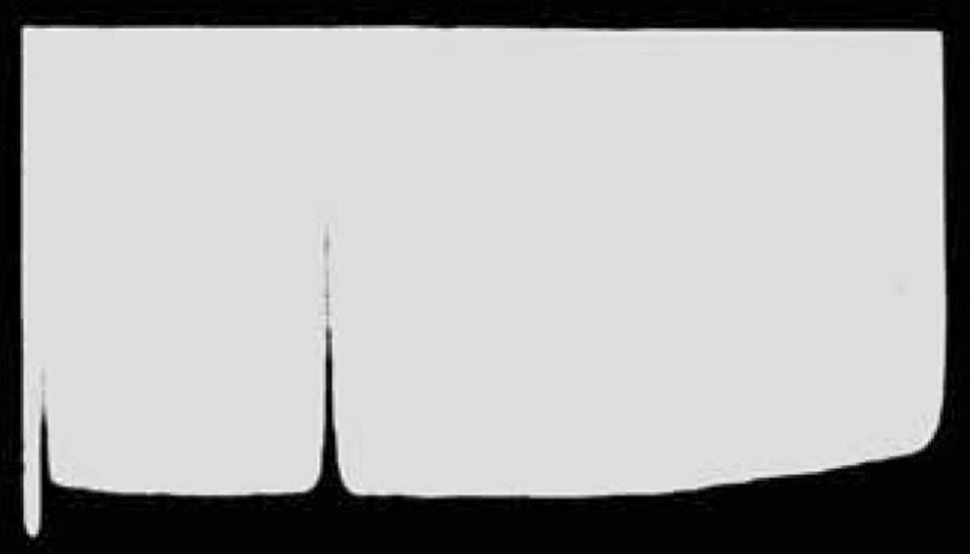
Schlieren profile from a Svedberg analytical ultracentrifuge showing a single hypersharp boundary for the preparation of calf-thymus DNA of high purity. The direction of sedimentation is from left to right Adapted from Cecil and Ogston (1948). The peak to the left is the air/solvent meniscus. Reproduced, with permission, from the Royal Society of Chemistry

Its use either directly or indirectly has been involved in some of the great discoveries over the last 100 years, including the establishment by Svedberg and Fåhraeus (1926) that proteins were discrete molecular entities of defined size and the discovery of the semi-conservative nature of the replication of DNA by Meselson and Stahl (1958). It was also used to establish the purity of DNA preparations (Gulland et al. 1947a; Cecil and Ogston 1948)—Fig. 1—used in the discovery of hydrogen bonds between the base pairs in DNA (Gulland et al. 1947b; Creeth et al. 1947) and the first technique to yield reliable molecular weight estimates for DNA (Cecil and Ogston 1948).

The unique property of being a true solution or “matrix free” technique (without immobilisation onto a surface or ionization/vaporisation) and possessing an inherent fractionation ability without the need of a separation matrix (column or membrane), and for molecular weight/size determination, without the need for calibration standards renders it an essential tool in macromolecular Biochemistry and Polymer Science. It can be used for the analysis of interactions in solution (self-interactions or “self-association” and macromolecular-ligand interactions) in terms of stoichiometries and reversibility (especially when the molecular weights of the interacting species are known precisely from mass spectroscopy), and when used in combination with other techniques such as viscometry, nuclear magnetic resonance, light scattering and X-ray scattering, can provide information about the overall conformation (shapes) of macromolecules in free solution (García de la Torre and Harding 2013).

We illustrate this diversity by reviewing the seminal role played by the analytical ultracentrifuge in recently published studies we have been involved with: (1) lignin-like isoeugenols (of molecular weights *M *= 0.4–0.9 kDa) of potential importance in the conservation of archaeological wood, (2) protein-like self-association of a functional amino-cellulose (*M* = 3.25 kDa), (3) dimerization of a small (*M* = 1.449 kDa) glycopeptide antibiotic vancomycin of current interest in the field of understanding the mechanisms behind antimicrobial resistance, (4) oligomerisation of tetanus toxoid protein (*M* = 150 kDa) and its incorporation into (5) two huge glycoconjugate vaccines with a very broad distribution of material with molecular weights up to 100 MDa. In illustrating the diversity, we will highlight developments in hydrodynamic analysis which have made the AUC a relevant technology. We conclude by pointing to a potential future development for extending its capability to highly concentrated systems, relevant, for example, for the characterisation of the behaviour of monoclonal antibodies at the high concentrations they are used for administration.

## Lignin-like isoeugenols (*M* ~ 0.4–0.9 kDa)

**Fig. 2:**
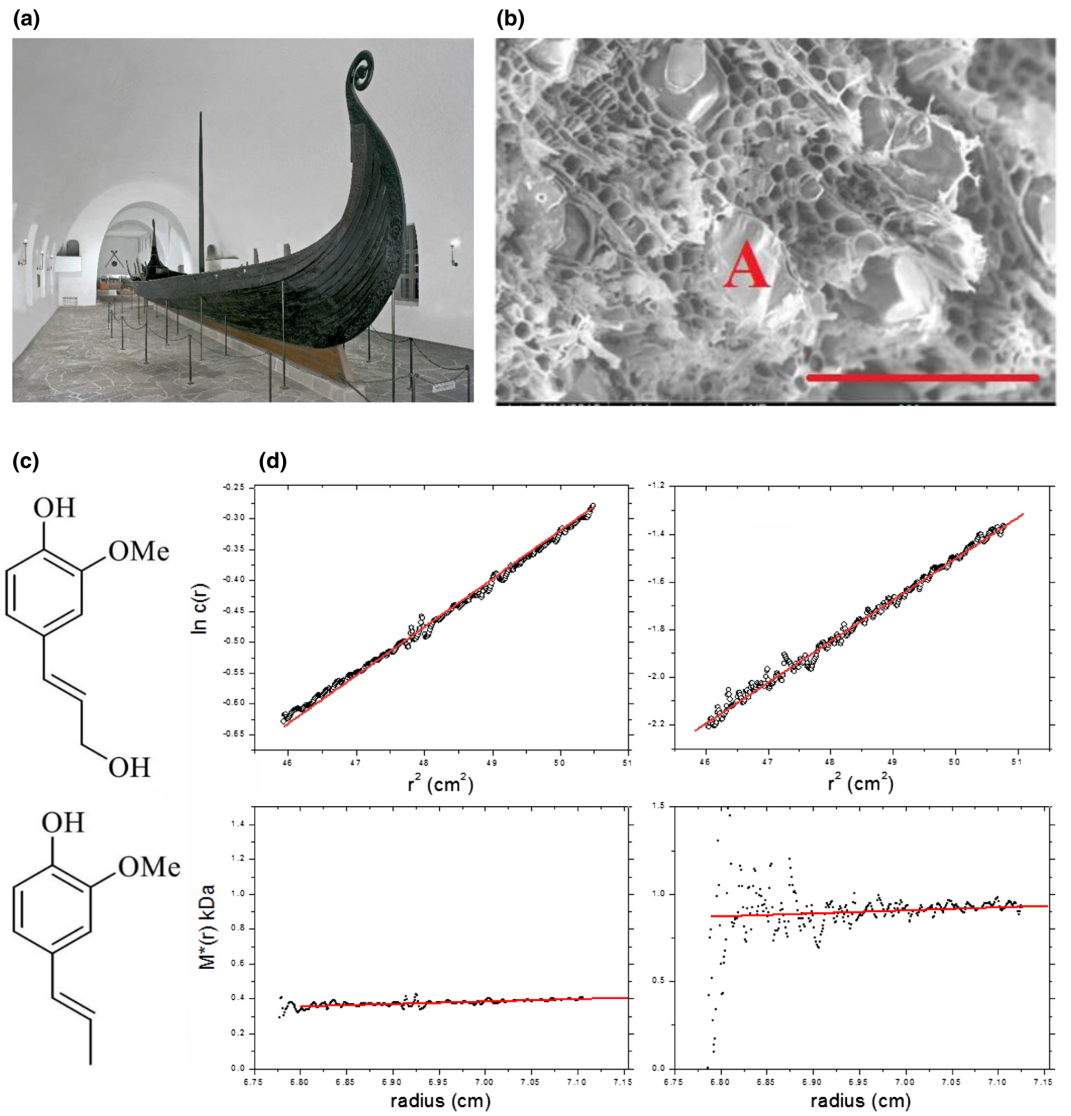
Consolidating alum-treated wood: the Saving Oseberg project. **a** The Oseberg Viking ship at the Skiphuset, Oslo. Artefacts discovered with the ship and displayed at the back of the hall, are now in danger of disintegration. **b** Scanning electron microscopy image of a piece of alum-treated wood. Scale bar = 200 μm. Empty fibrils are clearly seen, as are alum crystals (one is marked with an A). **c** Top conferyl alcohol, a monomer unit for lignin, and bottom, isoeugenol monomer unit. **d** concentration distribution and M* extrapolation to the cell base (right hand axis) to give *M*_w_, the weight average molecular weight for the whole distribution for (left) isoeugenol IE2, 0.4 kDa and (right) isoeugenol IE4, 0.9 kDa. **a**, **b** Courtesy of the Cultural History Museum, University of Oslo. **c**, **d** from McHale et al. (2017) and reproduced with permission of Nature Journals

Conserving archaeological wood is a major problem due to long-term decay processes involving in particular the cellulose and lignin. This is particularly true for wood that had been originally conserved by the application of hot alum or potassium aluminium sulphate—KAl(SO_4_)_2_·12H_2_O—which crystallizes in the wood preventing it from cracking on drying. Unfortunately, over long periods, the alum yields H_2_SO_4_ and this process has happened, for example, in the valuable Oseberg Viking ship artefacts in Oslo. The ship (Fig. 2a)—which has become a symbol of Norway’s national identity—had been discovered buried in blue-clay—an ideal anaeorobic preservative, a few km inland from the western banks of Oslo Fjord. Immediately after its excavation in 1904, the many fascinating objects found with the ship—a wagon, sledges, buckets, barrels and beds—were alum treated. Now, over 110 years later, there is unfortunately very little original cellulose and lignin material left—as the scanning electron microscopy image of Fig. 2b shows—placing the objects under serious threat. Replacement by ‘consolidant’ materials has to be found which are small enough to be administered and then to be capable of polymerisation into strong and stable polymer networks in situ (see http://www.khm.uio.no/english/research/projects/saving-oseberg/ and Harding 2017). The materials should be preferably administered in non-aqueous form so as to minimize dissolution of the remaining alum crystals.

Native lignins were characterized by our laboratory (Alzahrani et al. 2016) and we showed by sedimentation equilibrium in the analytical ultracentrifuge (performed in dimethylsulfoxide), analysed using the SEDFIT-MSTAR algorithm developed with P. Schuck (Schuck et al. 2014) based on the M* function of Creeth and Harding (1982) that lignins from different wood sources (“alcell” and “kraft”) had weight average molecular weights of ~ 20 kDa (19 kDa alcell and 25 kDa kraft). Analysis of the distributions of molecular weight using the MULTISIG algorithm (Gillis et al. 2013) showed a relatively broad distribution for the alcell compared with the kraft, and by combining the molecular weights with intrinsic viscosity data it was possible to show using the ELLIPS1 algorithm (García de la Torre and Harding 2013) that both adopted a discoid structure of aspect ratio ~ 30:1 consistent with previous work.

McHale et al. (2016, 2017) have been seeking to develop lignin replacements for the decayed wood using isoeugenols (monomer *M* ~ 160 Da, comparable to that of an amino-acid or carbohydrate residue) which are structurally very similar to lignins, built up from very similar monomer units (Fig. 2c), but of lower molecular weight (low enough to be absorbed into the wood), but have to be made to polymerize in situ using a peroxidase in the presence of hydrogen peroxide H_2_O_2_. In this early study, in situ polymerized materials in dimethyl sulfoxide (DMSO) were extracted and analysed using SEDFIT-MSTAR (Schuck et al. 2014) showing a moderate degree of association to give molecular weights (Fig. 2d) corresponding to ~ 3 and 6 mers (*M* ~ 400–900 Da); so clearly, this is a step in the right direction. More recently, the synthesis of polymers of *M* ~ 1600 Da has been achieved and further work is ongoing in the bid to extend the polymerization to 3–4000 Da.

## Amino-celluloses (*M* ~ 3.25–13 kDa)

**Fig. 3:**
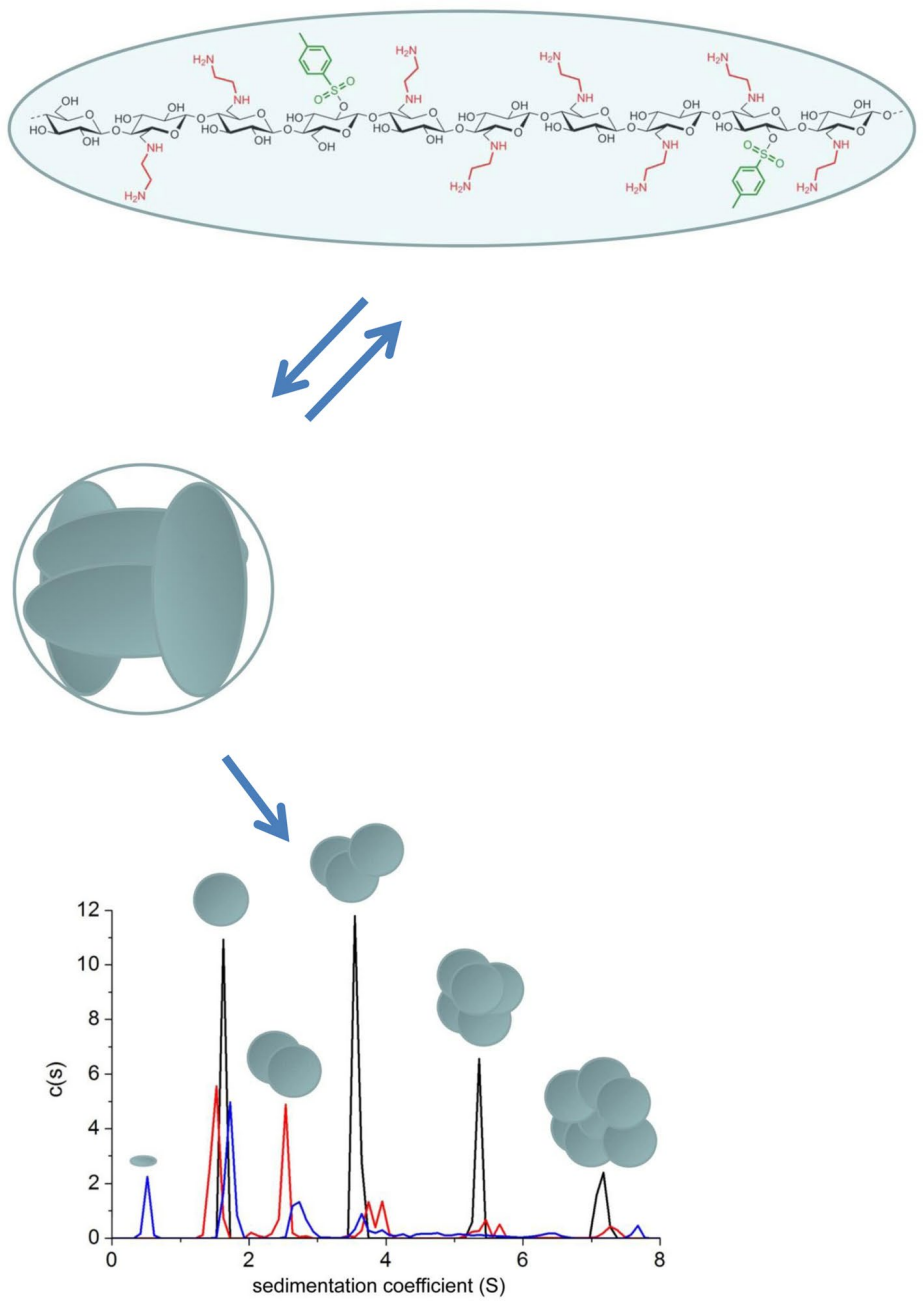
Reversible protein-like tetramerisation and further higher order association of amino-cellulose AEA-1. Top: monomer unit of degree of polymerization ~ 10, yielding an *M* ~ 3250 Da and *s* ~ 0.5 S. Middle: assembly into tetramers with *M* ~ 13,000 Da and *s* ~ 1.7 S. Lower: sedimentation coefficient distribution for AEA-1 at different concentrations 2.0 mg/ml (black), 1.0 mg/ml (red) and 0.75 mg/ml (blue). Based on the *s* ~ *M*^2/3^ scaling relationship the super-monomers associate into super-trimers, super-hexamers and super-9-mers with evidence also for some super-dimers, although the latter were not evident at the highest loading concentration. The proportion of the super-monomers drops relative to the higher order species showing partial reversibility even with the higher order association. From Nikolajski et al. (2014) and reproduced with permission of Nature Journals

Work is also underway to find suitable consolidant materials to replace the other main degraded component in archaeological wood—cellulose. One possibility is a group of synthetic amino-derivatives of celluloses whose monomer molecular weights seem in the range for penetration into wood and they have the ability to self-assemble into larger structures. “Amino-celluloses” were the subject of two recent studies using analytical ultracentrifugation (Heinze et al. 2011; Nikolajski et al. 2014). The first paper (Heinze et al. 2011) reported some unusual self-associative behaviour that was at least partially reversible. This was mainly on the basis of sedimentation velocity experiments which revealed multiple peaks following the *s *~ *M*^*b*^ power law “rule”, with *s* the sedimentation coefficient and the power law or ‘scaling’ coefficient *b* = 0.67, a value typical for globular proteins and very different from what is normally expected for polysaccharides, where *b* is normally between the limits of 0.15 (stiff rods) and 0.4–0.5 (random coils). The second paper (Nikolajski et al. 2014)—on the basis of sedimentation equilibrium measurements combined with sedimentation velocity—showed a two stage associative process with a monomer species of *M* ~ 3.25 kDa associating rapidly and reversibly into a tetrameric structure (*M* ~ 13 kDa) which then associated into higher order structures corresponding to the multiple sedimentation velocity peaks (Fig. 3). All measurements were done in aqueous solution. Such properties may prove useful as a cellulose replacement in decayed wood.

The related polycationic polysaccharide chitosan—a partially deacetylated form of chitin (poly-*N*-acetyl glucosamine) has also been considered. Here the starting materials are usually too large and the chitosans have to be depolymerised to molecular weights *M* < 4 kDa, and a recent study by Wakefield et al. (2018) successfully used a combination of UV radiation and hydrogen peroxide to bring the weight average molecular weight—as assessed by sedimentation equilibrium analysis—to within this range. Again, measurements were done in aqueous solution. The task is to find an amino-cellulose or chitosan of suitable molecular weight that is soluble in non-aqueous medium and with the desired properties to penetrate the wood and be capable of interacting with and reinforcing the fibrils within the wood, and repolymerisation within the wooden structure.

## Dimerization of the glycopeptide antibiotic vancomycin (*M* ~ 1.5 kDa) and its interaction with the membrane protein VanS (*M* ~ 47 kDa)

**Fig. 4:**
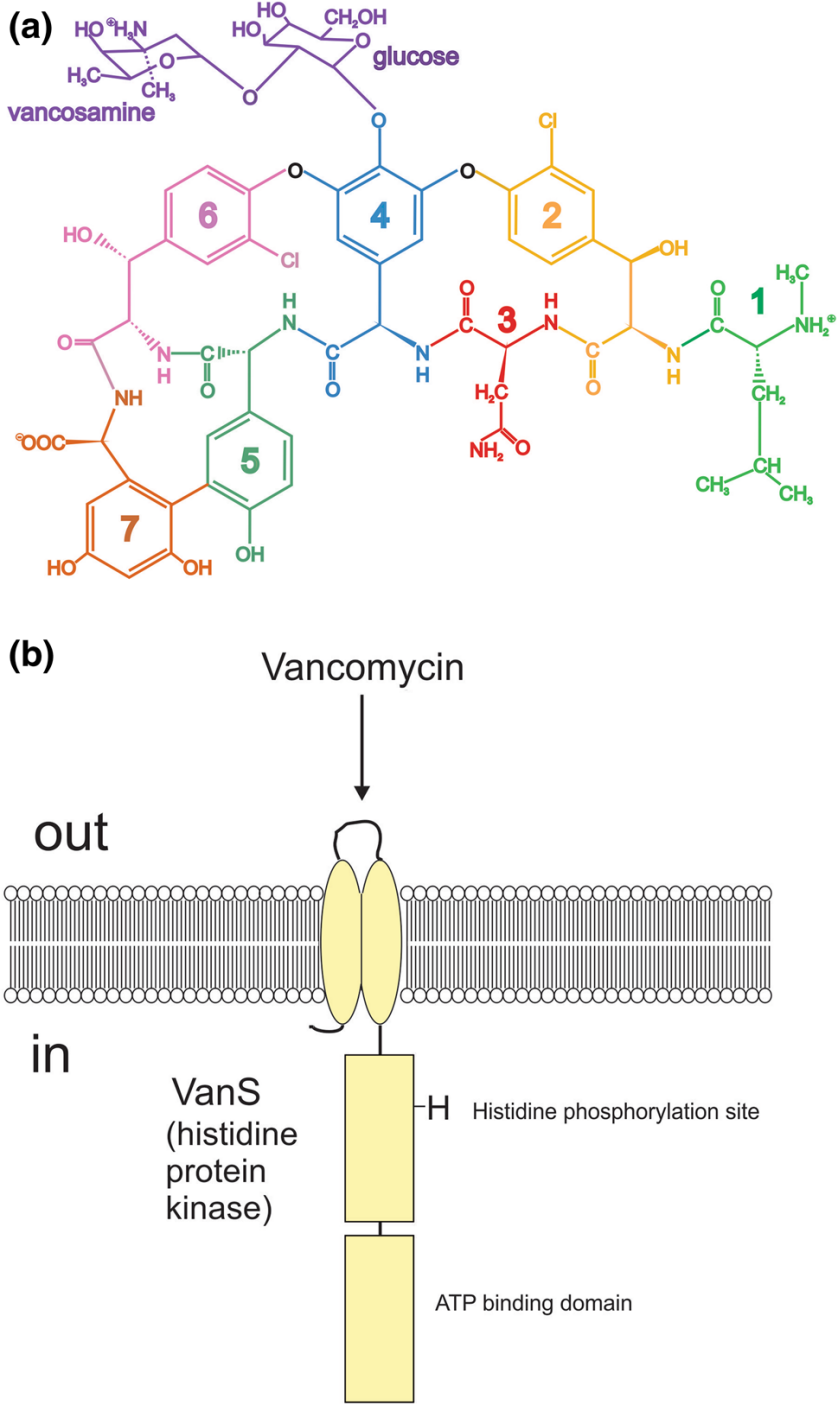
Schematic structures for (**a**) the glycopeptide antibiotic vancomycin and (**b**) the VanS membrane-bound sensor kinase involved in inducer sensing. Courtesy of Dr. M. K. Phillips-Jones

There is currently great interest in trying to elucidate the mechanisms of resistance in pathogenic bacteria to antibiotics and how this resistance can be permanently overcome (see, for example, Phillips-Jones and Harding 2018 and references therein). An important part of this is the study of the antibiotics used, the receptors for them and the nature of the ligand-receptor interaction. One such antibiotic that has been the subject of two recent publications (Phillips-Jones et al. 2017a, b) is the antibiotic vancomycin and its interaction with the VanS protein system (Fig. 4). Vancomycin is a small glycopeptide of monomer molecular weight 1449 Da (Fig. 4a) and VanS is a membrane protein of monomer molecular weight from mass spectroscopy of 47 kDa (Phillips-Jones et al. 2017a) with an extracellular domain, just two transmembrane regions and two large cytoplasmic domains (Fig. 4b), rendering the protein aqueous soluble.

**Fig. 5:**
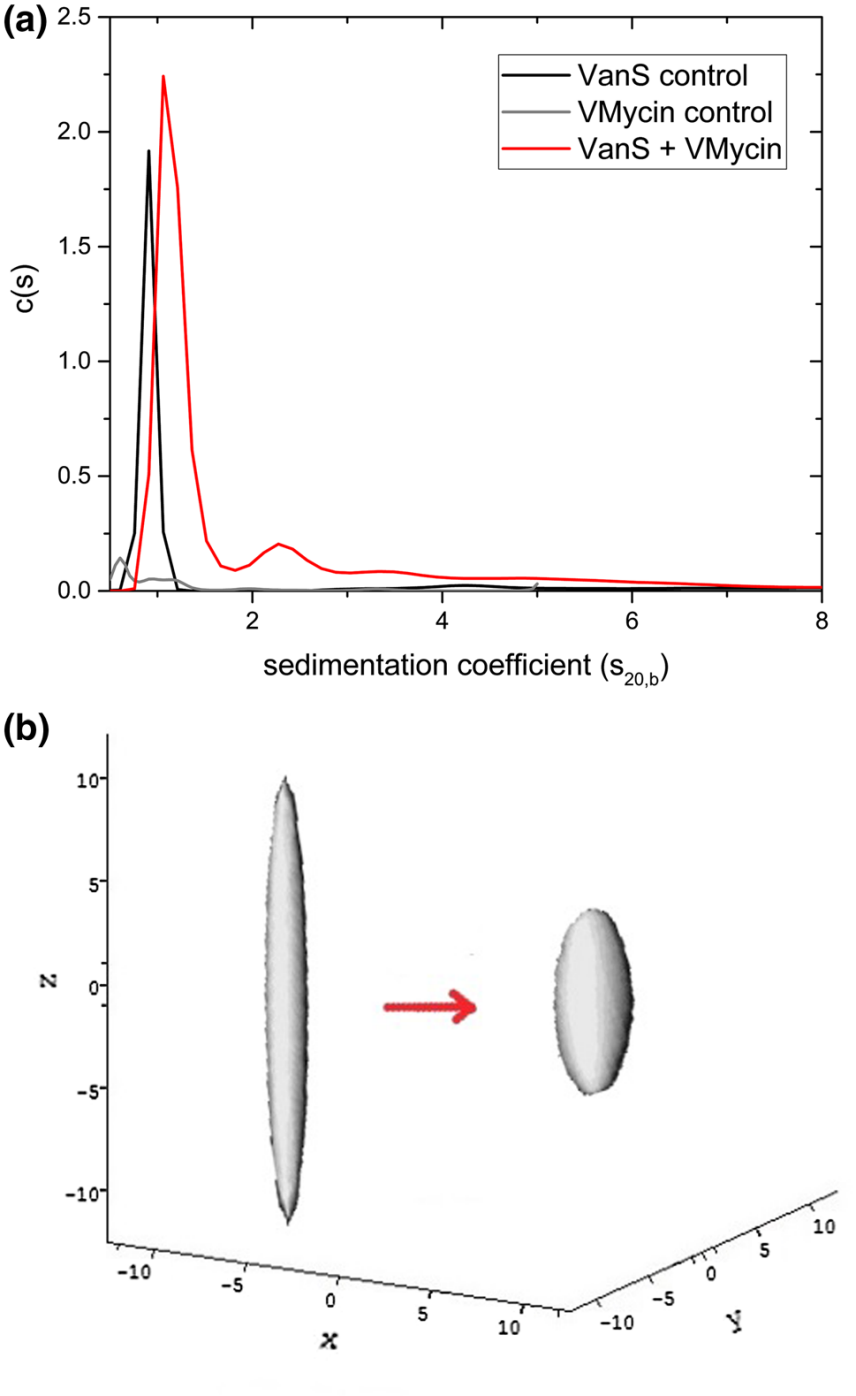
Hydrodynamics of VanS **a** sedimentation coefficient concentration distribution, c(s) vs s profile for VanS (black profile) in aqueous buffer pH ~ 7.9, *I* = 0.1 (supplemented with 20% glycerol) at 20.0 °C at a loading concentration of 0.25 mg mL^−1^ (5.4 μM). Also shown is the profile for vancomycin 0.019 mg mL^−1^ (12.8 μM) (grey profiles) and a mixture of VanS and vancomycin (red profile) under the same conditions, showing a 30% increase in the sedimentation coefficient for VanS, and **b** hydrodynamic shape of the VanS protein from ELLIPS1 in the absence (left) and presence (right) of vancomycin. The axial ratio reduces from ~ 12:1 to a more compact structure of axial ratio ~ 5:1 Adapted from Phillips-Jones (2017a, b) and reproduced with permission of Nature Journals

In the first paper (Phillips-Jones et al. 2017a), VanS was characterized by a number of techniques including sedimentation velocity and sedimentation equilibrium in the analytical ultracentrifuge. In aqueous solution (supplemented with 20% glycerol) VanS was found to be monomeric by sedimentation equilibrium analysed by both SEDFIT-MSTAR (Schuck et al. 2014) and MULTISIG (Gillis et al. 2013) analyses with a weight average molecular weight *M*_w_ of (47 ± 1) kDa, with some evidence of a small amount (~ 1%) of tetramer. The sedimentation coefficient distribution (Fig. 5a) obtained using SEDFIT (Dam and Schuck 2004) showed primarily a single species with a small amount of higher molecular weight species, presumably tetramer. Combination of the sedimentation coefficient, $s = \, \left( {0.9 \pm 0.1} \right){\text{S}}$ (corresponding to a value for $s_{{20,{\text{w}}}} = \, 2.3{\text{S}}$, after normalization to the density and viscosity of water at 20.0 °C) and molecular weight of the monomer, led to a high value for the Perrin translational frictional function *P* = (1.64 ± 0.09), which yielded using ELLIPS1 (García de la Torre and Harding 2013) a prolate axial ratio of ~ (12 ± 2). Intriguingly, the addition of vancomycin—whose own sedimentation coefficient is < 0.5S—led to a significant positive shift of the sedimentation coefficient by > 30% to $s = \, \left( {1.2 \pm 0.2} \right){\text{S}}$ (corresponding to an $s_{{20,{\text{w}}}} = \, 3.1{\text{S}}$ which could be either due to a ligand-induced dimerization of the VanS or due to a conformation change to a more compact conformation (of axial ratio ~ 5:1). As to which was the most likely of these two scenarios, this was addressed in the second paper (Phillips-Jones et al. 2017b) which demonstrated no significant change in the molecular weight of VanS caused by its interaction with vancomycin, i.e. it remained in the monomer state (again on the basis of both SEDFIT-MSTAR and MULTISIG analyses), and hence a ligand-induced conformation change (Fig. 5b) was the more likely explanation of the increase in the sedimentation coefficient. Yet, dimerization seems to be an essential general requirement for histidine kinase function (e.g. Gao and Stock 2009). So how could intact VanS be active in autophosphorylation assays and undergo a response (compaction in shape) upon vancomycin addition in the absence of any observed dimerization? One explanation is as follows (M. Phillips-Jones, *pers commun*). Although VanS is predominantly a soluble protein, it is nevertheless membrane-anchored in vivo by two transmembrane regions. In the absence of the native membrane (or detergent, as undertaken in these experiments), it is conceivable that intact VanS adopts a partially unfolded state, accounting for the extended conformation of 12:1 axial ratio. However, upon vancomycin addition (or also presumably the other ligand, ATP), VanS changes conformation and adopts its functional state, evidenced by the more compact shape adopted (~ 5:1 axial ratio). Refolding of proteins (in some cases to make dimers from monomers) and changes in conformational flexibility upon ligand binding have been documented previously (Stöckel et al. 1994, 1997; Celej et al. 2003; Moscoso et al. 2011), as of course has protein stabilisation in response to binding by certain ligands (e.g. Toleikis et al. 2012; Allison et al. 2015; Mazal et al. 2018). Therefore, it is conceivable that dimerization of VanS occurs in the presence of the ATP ligand in the autophosphorylation assays whilst monomeric VanS is sufficient to show a conformational change in the presence of vancomycin. All of the data in Phillips-Jones (2017a, b) support this explanation and highlight the promise of using recombinant intact VanS for ligand interaction studies.

**Fig. 6:**
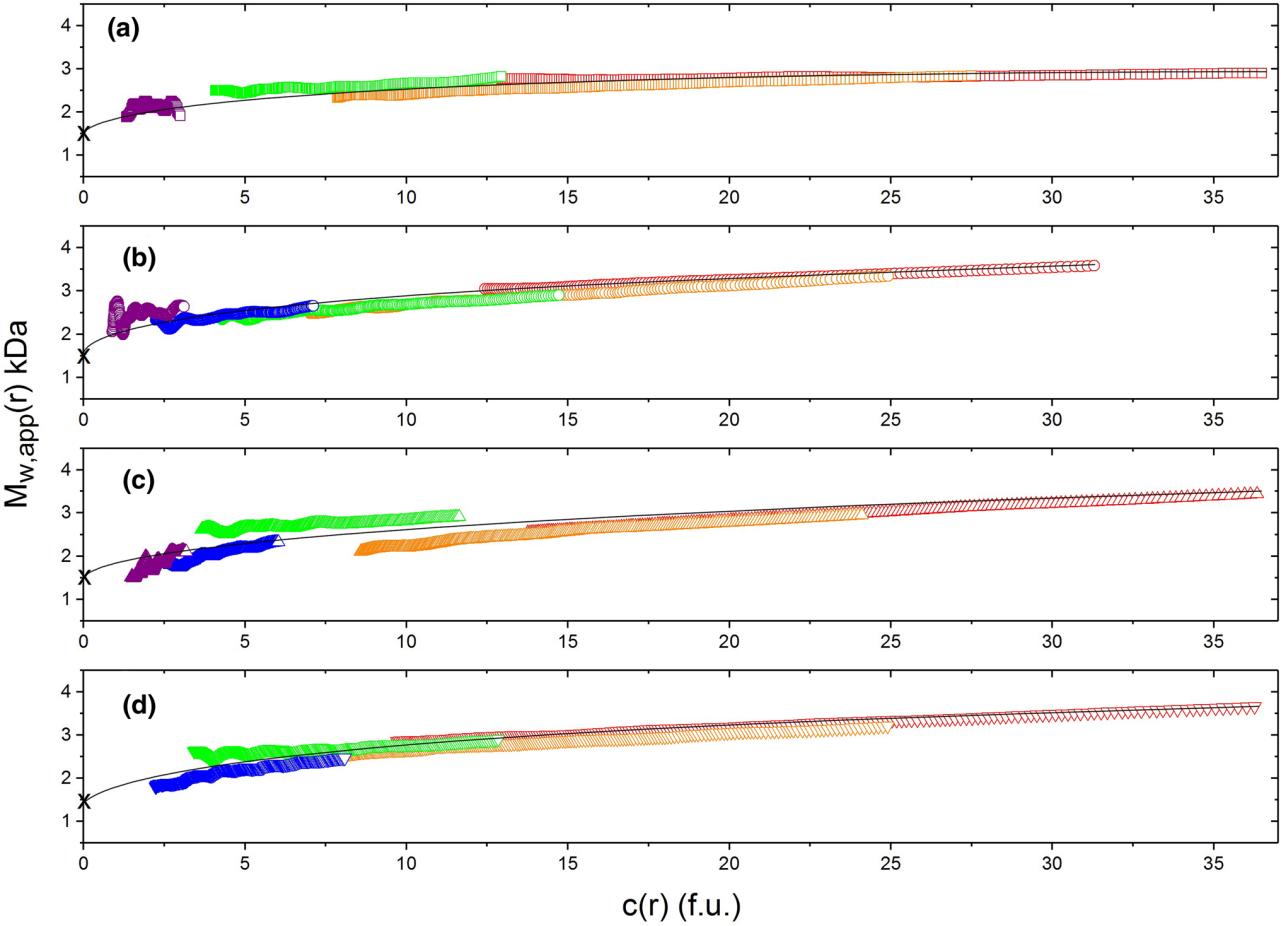
Diagnostic sedimentation equilibrium plots confirming a completely reversible dimerization for vancomycin under different aqueous solvent conditions (supplemented with 20% glycerol). *M*_w_(*r*) plotted against local concentration *c*(*r*) in interference fringe units from SEDFIT-MSTAR for different concentrations: violet (0.625 mg mL^−1^), blue (1.25 mg mL^−1^), green (2.5 mg mL^−1^), orange (5.0 mg mL^−1^) and red (10.0 mg mL^−1^). **a** 10 mM HEPES. **b** 10 mM HEPES + 100 mM NaCl. **c** 10 mM HEPES + 100 mM NaCl + 20% glycerol. **d** 0.9% NaCl in deionised, distilled water. Overlap of the plots confirms reversibility. Reproduced with permission from Nature Journals

Phillips-Jones et al. (2017b) also provided a detailed examination of the dimerization equilibrium behaviour of vancomycin, again using SEDFIT-MSTAR, with a range of solvent conditions and loading concentrations. Besides, the weight average molecular weights *M*_w_ over the whole distribution, SEDFIT-MSTAR also gives the weight average molecular weight values *M*_w_(*r*) at individual radial positions in the ultracentrifuge cell. For each of the four solvent conditions: 10 mM HEPES; 10 mM HEPES + 100 mM NaCl; 10 mM HEPES + 100 mM NaCl +20% glycerol; 0.9% (w/v) NaCl in deionised, distilled water, overlap of point weight average molecular weights *M*_w_(*r*) vs local concentration in the centrifuge cell *c*(*r*), where *r* is the radial position, plotted for different loading concentrations confirmed a fully reversible dimerization process, with some evidence of further association (Fig. 6). Classical Kegeles and Rao (1958) analysis (see also Kim et al. 1977) of the sedimentation equilibrium data showed that the vancomycin dimerization was a relatively weak one, with molar dissociation constants ranging from 35 to 50 μM across the range of solvent conditions. All the solvents used were aqueous: the VanS system is, however, complex with the large cytoplasmic domain commensurate with aqueous solvent conditions, but possesses also a significant transmembrane domain which is more commensurate with an appropriate detergent as solvent, such as n-dodecyl β-d-maltoside (DDM). Follow-up work is underway exploring if this behaviour (VanS monomer, weak interaction with vancomycin, and weak, fully reversible dimerization of the vancomycin itself)—all done under aqueous solvent conditions (i.e. more commensurate with the cytoplasm) are reproduced under the non-aqueous conditions of the membrane. This will give us a better understanding of this particular component of an antimicrobial resistance “nanomachine”(Phillips-Jones and Harding 2018).

## Dimerisation of the tetanus toxoid protein (*M* ~ 150 kDa)

**Fig. 7:**
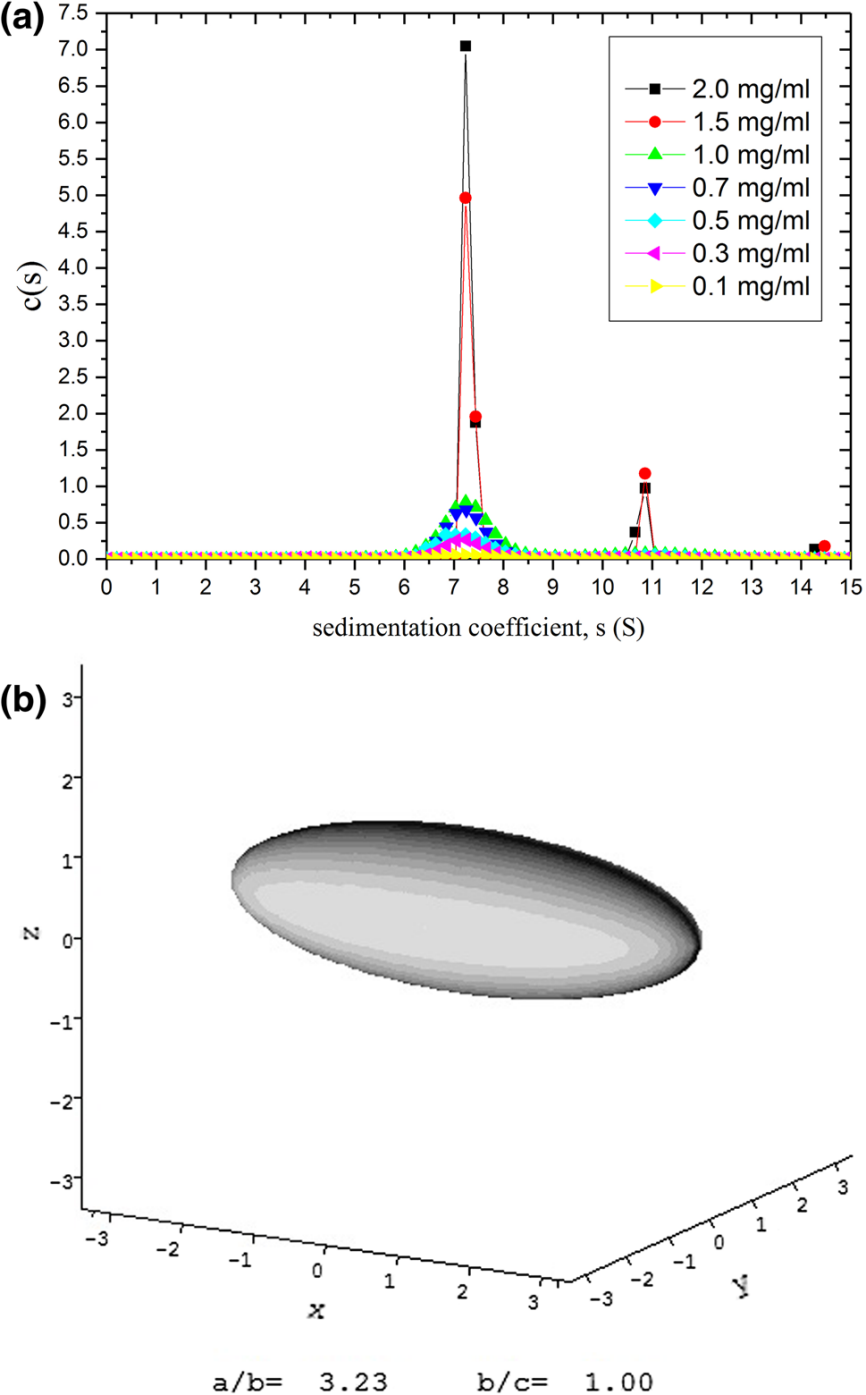
Tetanus toxoid protein—used in glycoconjugate vaccines to help stimulate T-cell-mediated long-lasting responses. **a** Sedimentation coefficient distribution from SEDFIT analysis for seven loading concentrations showing ∼ 14% of dimer, **b** prolate ellipsoid representation using ELLIPS1 analysis for monomeric tetanus toxoid protein showing an asymmetric structure of axial ratio ∼ 3. The slightly elongated structure facilitates the conjugation with polysaccharide chains. From Abdelhameed et al. (2012) and reproduced with permission from Elsevier

Tetanus toxoid protein is one of the main proteins used for conjugation with capsular bacterial polysaccharides in the construction of glycoconjugate vaccines against pathogenic bacteria such as *Neisseria meningitidis* and *Haemophilus influenzae* b (Astronomo and Burton 2010) and help to give a long-lasting T-cell-based effect for vaccines. We recently showed (Abdelhameed et al. 2012) by combining sedimentation velocity (Fig. 7a) with sedimentation equilibrium analysis that TTP was mostly monomeric in solution with ~ 14% dimer. Both the Perrin frictional function *P* and the viscosity increment *ν* (obtained from intrinsic viscosity measurement) point to an asymmetric structure: ELLIPS1 analysis indicates a prolate structure of aspect ratio of ~ 3:1 (Fig. 7b) coincidentally like the cartoon shape given earlier by Astronomo and Burton (2010). This asymmetric shape provides a greater surface area for conjugation with the relevant polysaccharides, making it a popular choice for researchers.

## Capsular polysaccharides from *Haemophilus influenzae* type b (weight average *M*_w_ ~ 1200 kDa) and glycoconjugates (*M*_w_ ~ 7300 kDa) with TTP

**Fig. 8:**
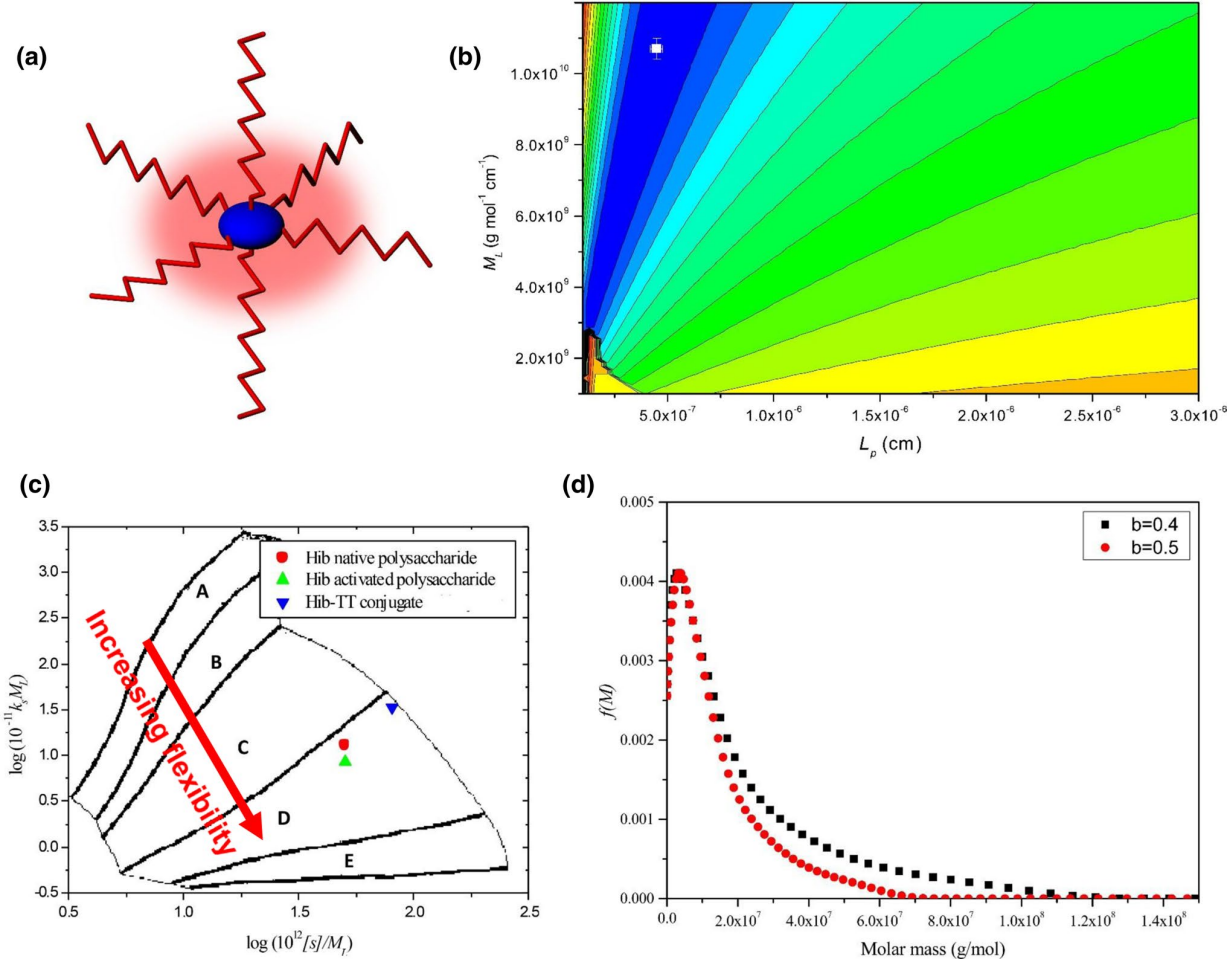
Characterisation of the glycoconjugate Hib PRP-TT **a** schematic structure showing the asymmetric tetanus toxoid protein (in blue) with polysaccharide chains (in red) attached. **b** HYDFIT plot. Combination of viscosity-molecular weight and sedimentation coefficient-molecular weight data, and the respective Bushin–Bohdanecky and Yamakwa–Fuji relations to yield a target function which when minimized (shown as a white square on the contour map) gives the best fit estimates for the persistence length *L*_p_ (and mass per unit length). The value obtained for *L*_p_ = ∼ 4.5 × 10^−7^ cm corresponding to a random coil conformation, similar to that for the unconjugated polysaccharide and activated (with linker ready for conjugation) polysaccharide. **c** conformation zoning plot: unconjugated polysaccharide, activated polysaccharide and glycoconjugate are all zone D (random coil). **b**, **c** confirm that it is the randomly coiled polysaccharide which dominates the hydrodynamic properties of the glycoconjugate and provides a time-averaged blanket around the protein (shown in red haze in **a**). **d** Molecular weight distribution *f(*M) profile from the Extended Fujita method (Harding et al. 2011) for Hib PRP-TT conjugate, showing a broad distribution of weight average *M*_w_ ~ 7300 kDa and with a small fraction of species in the distribution appearing to reach ~ 100  MDa From Abdelhameed et al. (2016a). Reproduced with permission from Nature Journals

Isolated polysaccharides from *Haemophilus influenzae* type b or “Hib” have been used as vaccines against influenza, although the response is generally short lived. Coupling to TTP or other proteins which stimulate the involvement of T cells—shown schematically in Fig. 8a—produces a much longer lasting effect. The polysaccharides themselves are very large as determined by sedimentation equilibrium in the analytical ultracentrifuge (weight average molecular weight *M*_w_ ~ 1200 kDa), and these and their TTP glycoconjugates (*M*_w_ ~ 7300 kDa) were the subject of a recent study by ourselves (Abdelhameed et al. 2016a). This was followed by a similar study on polysaccharides and glycoconjugates from *N. meningitidis* (Abdelhameed et al. 2016b). Besides addressing important issues concerning molecular weight and molecular weight distribution for these very large and heterogeneous systems, these studies also sought to address whether the conformation of the glycoconjugates was affected more by the protein or polysaccharide moiety, factors which can have a large impact on the stability of formulations.

The polysaccharides from both bacteria were shown to adopt flexible coil conformations with low persistence lengths *L*_p_ < 10 nm on the basis of a global minimization method known as HYDFIT developed by Ortega and García de la Torre (2007). HYDFIT involves the minimization of a target function ∆ for *L*_p_ and the mass per unit length *M*_L_ based on the Yamakawa–Fuji relations linking the sedimentation coefficient with molecular weight and corresponding Bohdanecky relations linking the intrinsic viscosity with molecular weight (Fig. 8b). Similar flexible coil conformations with low persistence lengths were also found for the polysaccharides activated with a cross linker for conjugation—and also their glycoconjugates with TTP—showing that they adopted the conformation properties of the polysaccharides and not the TTP. This was confirmed by a method known as conformation zoning (Fig. 8c), which involves the combination of the sedimentation coefficient and Gralen coefficient *κ*_s_ (from the concentration dependence of *s*) developed by Pavlov et al. (1997). The combined *s* and *κ*_s_ values are only commensurate with a random coil conformation, confirming the HYDFIT results (Fig. 8b).

To obtain the molecular weight distribution, a method we developed with P. Schuck known as the Extended Fujita method was employed (Harding et al. 2011). The method transforms a sedimentation coefficient distribution g(*s*) vs *s* into a molecular weight distribution *f*(*M*) vs *M* using the following transformation equations:

5 $$f\left( M \right) = ({\text{d}}s/{\text{d}}M) \cdot {\text{ g}}(s)$$

with

6 $$M = \, (s/\kappa_{s} )^{1/b}$$

and

7 $${\text{d}}s/{\text{d}}M = b \cdot \kappa_{s}^{1/b} \cdot s^{{\left( {b - 1} \right)/b}}$$

*b* is the scaling coefficient (for a flexible coil this will be between 0.4 and 0.5) and κ_*s*_ can be found from Eq. (6) provided that at least one value of *M* (e.g. *M*_w_ from sedimentation equilibrium is known for one value of *s* (e.g. the weight average *s* value). The algorithm has been incorporated into the SEDFIT programme of Schuck (Dam and Schuck 2004). Figure 8d shows the distribution for Hib-TTP glycovaccines for the two possible values of *b*. The large distribution of molecular weight is evident with a weight average *M*_w_ of ~ 7300 kDa and some material appearing to reach up to 100 MDa. Very similar behaviour was found for the glycoconjugates formed from conjugation of TTP with capsular polysaccharides from *N. meningitidis* (Abdelhameed et al. 2016b).

## Future trends—concentrated systems

I hope I have been able to provide a snapshot of the great diversity of sizes and types of substance that can be successfully characterised by the analytical ultracentrifuge. The examples I have focused on have all referred to dilute solution conditions, and these are well within the capabilities of the current optical systems available on commercially available instrumentation, most notably the Beckman-Coulter Optima XL-I analytical ultracentrifuge with its dual system of UV absorbance and Rayleigh interference. In my presentation I have not really touched on concentrated systems, and this is becoming increasingly important particularly in the field of monoclonal antibody research, a key area for drug development. We have been involved in antibody research for over three decades. This started with collaborations with researchers at USB Celltech and Prof. Dennis Burton’s group at the University of Sheffield and then the Scripps Institute at La Jolla. Using bead modelling developed by J. García de la Torre and co-workers, the first hydrodynamic model for IgE was produced correctly predicting the cusp shape conformation (Davis et al. 1990) and by 2007 papers were appearing (see e.g. Nobbmann et al. 2007; Lu et al. 2008) reporting the use of the analytical ultracentrifuge for assessing the stability of antibody preparations to processing (freeze–thaw and storage at elevated temperature), and in conjunction with other techniques such as dynamic light scattering (Nobbmann et al. 2007). Now the focus is very much on concentrated systems because of the high concentrations (80 mg/ml and higher) that are considered for administration. Unfortunately, these concentrations are out of reach of the current commercially available optical systems, although, oddly, the Schlieren (refractive index gradient) optical system—available in the original Svedberg analytical ultracentrifuge (Lloyd 1974) may be useful in this regard. Figure 1 shows such an image obtained with this system and the famous Beckman Model E had this—and concentrations > 80 mg/ml were possible. There may be a case for reintroduction of this type of system. The ability to measure at such high concentrations comes at a price, and issues of thermodynamic (or hydrodynamic) non-ideality caused by co-exclusion and polyelectrolyte effects can become serious. In the past, non-ideality phenomena were sometimes seen as advantageous as it was possible to use the second thermodynamic virial coefficient *B* to estimate the triaxial shape of a macromolecule using relations worked out by Rallison and Harding (1985). Indeed if *B* could be accurately calculated from knowledge of the overall structure of a macromolecule, then it could be eliminated as a variable in the thermodynamic equations—and this was made possible via the COVOL programme (Harding et al. 1999; Harding 2013). Finding corresponding relations for the equivalent hydrodynamic non-ideality coefficients at high concentration is still a major challenge.
